# Supplementary material for: Impact of enzymatic digestion on bacterial community composition in CF airway samples
Source: PeerJ. 2017 May 30;5:e3362. doi: 10.7717/peerj.3362 (PMC5452939; doi:10.7717/peerj.3362)
Supplement: Supplemental Information 1 [file peerj-05-3362-s001.pdf]

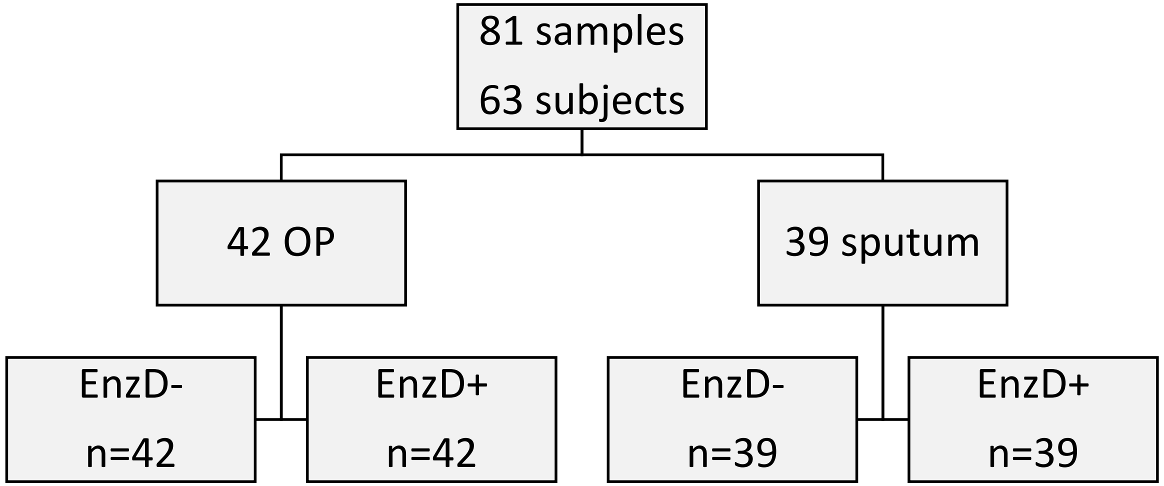

Figure S1: Study Design

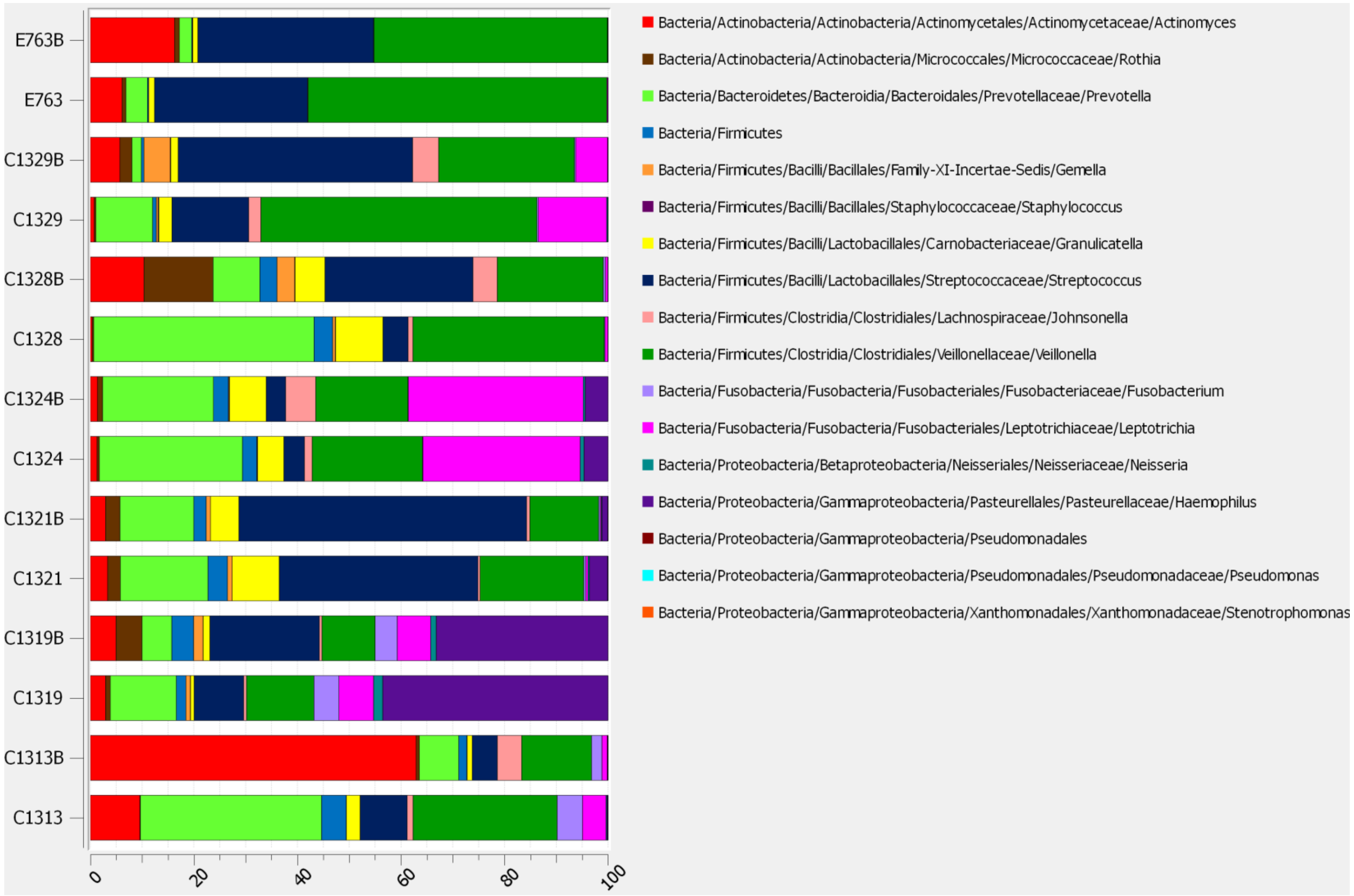

Figure S2: bacterial community composition for 7 culture negative samples. Samples ending with 'B' correspond to those with EnzD.

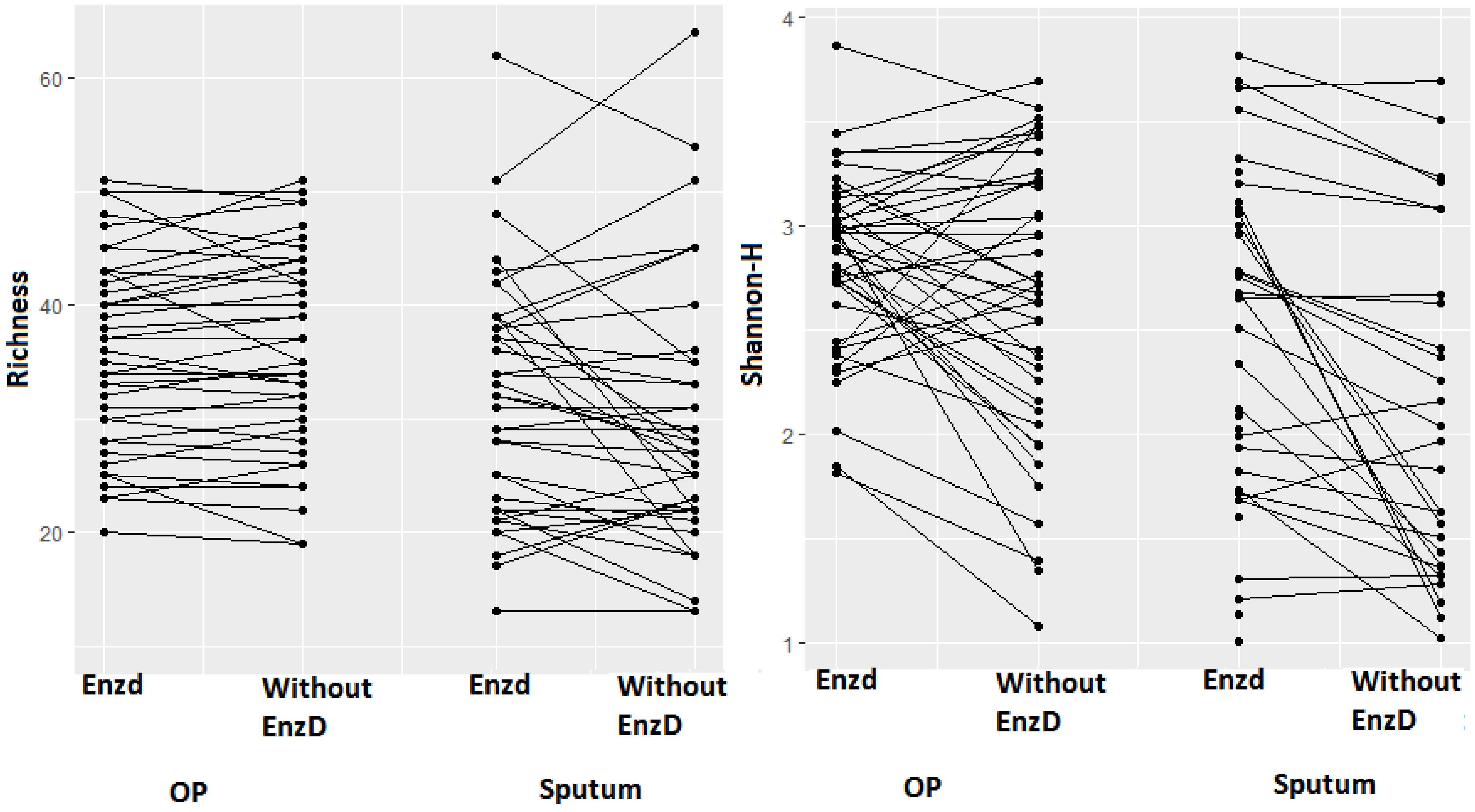

Figure S3: Changes in alpha diversity, richness (left) and Shannon H (right) between EnzD and nonEnzD for OP and sputum samples.

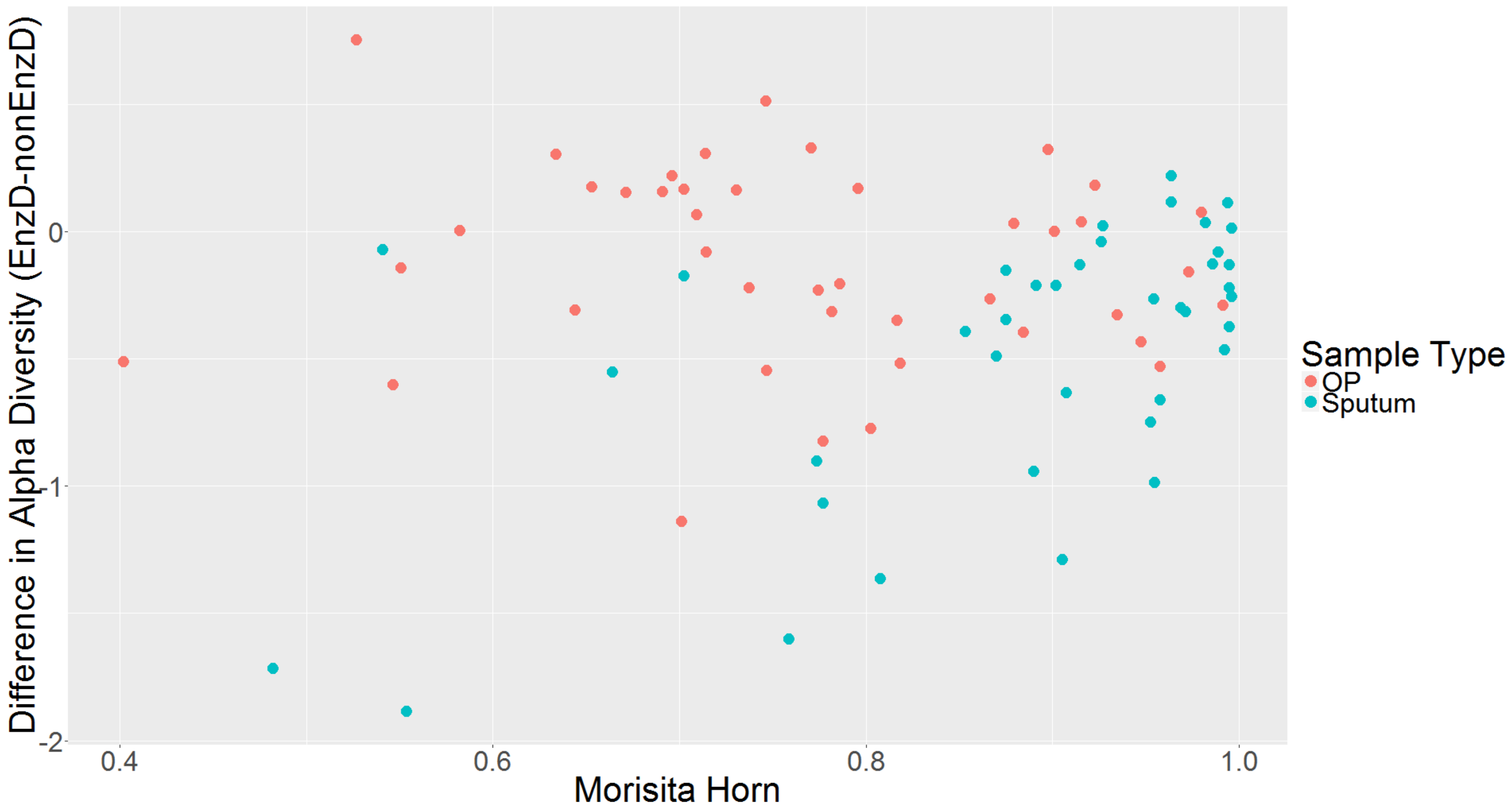

Figure S4: Changes in alpha diversity (EnzD-nonEnzD) versus MH. Positive values along the y-axis represent higher diversity without digestion.

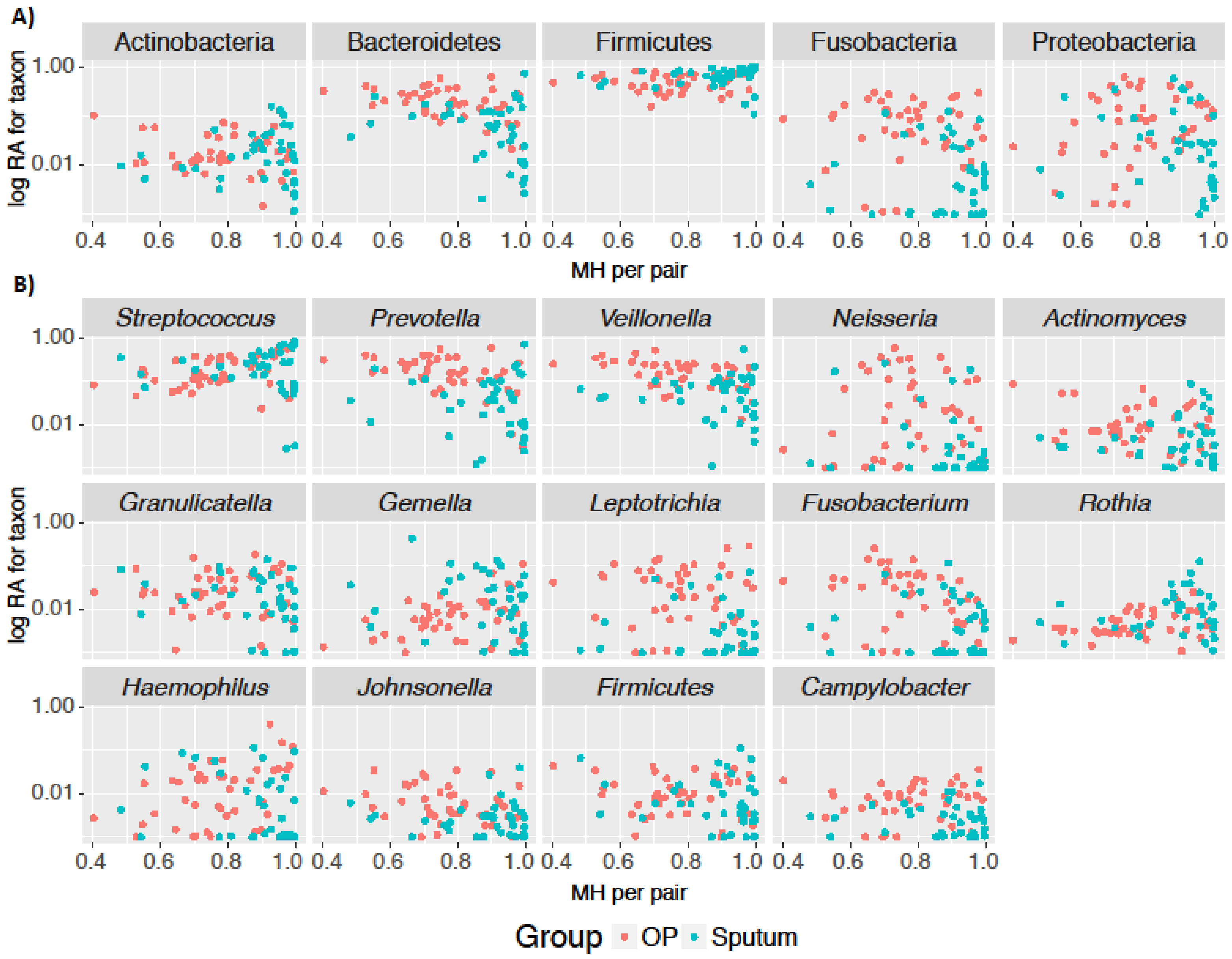

Figure S5: Scatterplots of relative abundance in non-EnzD sample versus MH of paired samples for specific phyla (left) and genera (right). OP samples are shown in red and sputum in blue
