# Supplementary material for: Impact of enzymatic digestion on bacterial community composition in CF airway samples
Source: PeerJ. 2017 May 30;5:e3362. doi: 10.7717/peerj.3362 (PMC5452939; doi:10.7717/peerj.3362)
Supplement: Supplemental Information 2 [file peerj-05-3362-s002.pdf]

```
#Load Libraries
library("vegan")
library(exactRankTests)
library(ggplot2)
library(reshape2)
library("Matrix")
library("diagonals")
library(schoolmath)
library(RGraphics)
library(gridExtra)
library("grid")
library("xtable")





#Read in Data
mypath<-"C:/Users/wkayla/Desktop/Microbiome/Enzyme Digest/Data/EnzymeDigest_Workspace_1_OTU.txt"
data=read.table(mypath, header = T, sep="\t",na.strings= "na")



#Code for Functions Used throught out this program
    #format Data From Explicet for use in R
    otu_from_explicet=function(data){
      colnames=data[,1]
      Id=colnames(data)[-1]
      data=t(data)
      colnames(data)=colnames
      data=data[-1,]
      data=apply(apply(data,2,as.character),2,as.numeric)
      data=cbind.data.frame(Id,data)
      rownames(data)=seq(nrow(data))
      return(data)}


    #Used for splitting taxa names
    name.split<-function(names){
      names=names
      save=strsplit(names,"/")

      h=0
      for(i in 1:length(names)){
        h[i]=length(save[[i]])}

      i=0
      name.list=NULL
      for(i in 1:length(save)){
        name.list[i]=save[[i]][h[i]]}
      return(name.list)
    }

    #Creates a block diagonal matrix
    rblock<- function(nb) {
      .bdiag(replicate(nb, {
        Matrix(c(0,1,1,0), 2,2) }))
    }
#Format and Clean Data

    #Transform data into nXp otu table
    otu=otu_from_explicet(data)

    #save Pseudmonas and Pseudmonasdales
    pseudmonasdales=otu$'Bacteria/Proteobacteria/Gammaproteobacteria/Pseudomonadales'
    pseudmonoas=otu$'Bacteria/Proteobacteria/Gammaproteobacteria/Pseudomonadales/Pseudomonadaceae/Pseudomonas'

    #Combine Pseudmonas and Pseudmonasdales into PseudomansPlus
    PseudomonasPlus=pseudmonasdales+pseudmonoas
    otu$PseudomonasPlus=PseudomonasPlus

    #Delete psedumonas and pseudomonasdales out of OTU
    otu=otu[,-which(colnames(otu)=="Bacteria/Proteobacteria/Gammaproteobacteria/Pseudomonadales/Pseudomonadaceae/Pseudomonas" )]

    otu=otu[,-which(colnames(otu)=="Bacteria/Proteobacteria/Gammaproteobacteria/Pseudomonadales")]

    #Create a dataframe of Relative Abundances
    rel.otu=otu[,3:255]/otu[,2]

    #create a data frame for use in Wilcox Exact Signed Rank Tests
    ##Delete out the Unclassified Taxa as well as Taxa whose lowest classification is Bacteria
    wilx.otu=otu[,2:255]

    #adjust the root
    wilx.otu$root=wilx.otu$root-wilx.otu$Unclassified
    wilx.otu$root=wilx.otu$root-wilx.otu$Bacteria

    #delete Unclassified
    un.row=which(colnames(wilx.otu)=="Unclassified" )
    wilx.otu=wilx.otu[,-un.row]

    #delete bacteria
    bac.row=which(colnames(wilx.otu)=="Bacteria" )
    wilx.otu=wilx.otu[,-49]

    #Recalculate Relative Abundance
    wilx.rel.otu=wilx.otu[,2:252]/wilx.otu[,1]


    #Create a Factor Variable for Digest; those ending in B have been digested all else were undigested
    digest=factor(rep(0:1,81),levels=c(0,1),labels=c("Without EnzD","With EnzD"))


    #Do the same for location of sample; those with C in the
    #original data are OP samples those  beginning with E are sputum samples
```

```
    area=factor(c(rep(0,84),rep(1,78)),levels=c(0,1),labels=c("OP","Sputum"))



    #Subset Data based on location of samples
    OP.otu=otu[1:84,]
    Sputum.otu=otu[85:162,]

    rel.OP.otu=rel.otu[1:84,]
    rel.Sputum.otu=rel.otu[85:162,]

    #rows that are(not) of EnzD samples
    digest_rows=seq(0,162,2)
    nondigest_rows=seq(1,162,2)

########################################### Alpha Diversity ###########################################

  #Shannon Alpha Diversity
  div=diversity(rel.otu,index ="shannon")
  boxplot(div~digest, data =otu, main ="Shannon Diversity Digest Versus Non")
  wilcox.exact(div~digest, paired=T)

      #Species Richness vs. Diversity
          #Begin with Shannon-H diversity
          H=diversity(rel.otu,index ="shannon")
          ## Species richness (S) and Pielou's evenness (J): From Vegan documentation
          S <- specnumber(rel.otu)
          J <- H/log(S)




  #OP Only Shannon Diversity
  boxplot(div[1:84]~digest[1:84], data =otu, main ="Shannon Diversity Digest Versus Non--OP")
  wilcox.exact(div[1:84]~digest[1:84], paired=T)

  #OP Only richness
  boxplot(S[1:84]~digest[1:84], data =otu, main ="Richness Digest Versus Non--OP")
  wilcox.exact(S[1:84]~digest[1:84], paired=T)

  #OP Only Peilou's evenness
  boxplot(J[1:84]~digest[1:84], data =otu, main ="Evenness Digest Versus Non--OP")
  wilcox.exact(J[1:84]~digest[1:84], paired=T)


  #Sputum Only Shannon Diversity
  boxplot(div[85:162]~digest[85:162], data =otu, main ="Shannon Diversity Digest Versus Non--Sputum")
  wilcox.exact(div[85:162]~digest[85:162], paired=T)

  #Sputum Only richness
  boxplot(S[85:162]~digest[85:162], data =otu, main ="Richness Digest Versus Non--Sputum")
  wilcox.exact(S[85:162]~digest[85:162], paired=T)

  #Sputum Only Peilou's evenness
  boxplot(J[85:162]~digest[85:162], data =otu, main ="Peilou's Evenness Digest Versus Non--Sputum")
  wilcox.exact(J[85:162]~digest[85:162], paired=T)


  #Extract only those realtive abundances whose median is at least 1% for OP samples
  medt=round(apply(rel.OP.otu,2,median),2)
  medt=medt[medt >=.01]
  pcoa.OP.otu=OP.otu[,names(medt)]

########################################### Beta Diversity ###########################################
  #PCoA for OP samples only
  dist=vegdist(pcoa.OP.otu,method="morisita")
  pcoa=cmdscale(dist)

  #Create a name list for Graphical use
  names=as.character(colnames(pcoa.OP.otu))
  name.list=name.split(names)

  #Create a data frame of varaibles from PCOA
  vars=pcoa.OP.otu
  colnames(vars)=name.list


  #add on the PCoA scores to the OP otu dataset
  ds=cbind(pcoa.OP.otu,pcoa)
  colnames(ds)[15:16]=c("m1","m2")

  #create vectors for those relative abundances whose pvalues are less than .005 for correaltion
  vec.sp<-envfit(pcoa, vars)
  vec.sp.df<-as.data.frame(cbind(vec.sp$vectors$arrows*sqrt(vec.sp$vectors$r),vec.sp$vectors$pvals))
  vec.sp.df$species<-rownames(vec.sp.df)
  colnames(vec.sp.df)<-c("Dim1","Dim2","Pvals","species")
  vec.sp.df<-vec.sp.df[vec.sp.df$Pvals<.005,]


  #create a biplot for OP samples
  r=rep(1:42,each=2)
  g2<-ggplot(ds,aes(x=ds$m1,y=ds$m2,group=r,color=digest[1:84]))+
            geom_line(col="black",linetype="dotted")+
            geom_point(size=3)+
            guides( col=F, size=F)+
            labs(x="Component 1", y="Component 2")+
            theme(text = element_text(size=20))+
            geom_segment(data=vec.sp.df,aes(x=0,xend=Dim1,y=0,yend=Dim2),
                         arrow = arrow(length = unit(0.5, "cm")),
                         colour="black",
                         stat="identity",inherit.aes = FALSE) +
            geom_text(data=vec.sp.df,aes(x=Dim1,y=Dim2,label=species),
                      inherit.aes = FALSE,
                      size=5)+
            coord_fixed()+
            xlim(-1,1)+
```

```
            ylim(-1,1)



  #extract only those bacteria whose relative abundance is at least 1% for sputum samples
  meds=round(apply(rel.Sputum.otu,2,median),2)
  meds=meds[meds >=.01]
  pcoa.Sputum.otu=Sputum.otu[,names(meds)]


  #PcoA for Sputum
  dist=vegdist(pcoa.Sputum.otu,method="morisita")
  pcoa=cmdscale(dist)

  #namelist from pcoa
  names=as.character(colnames(pcoa.Sputum.otu))
  name.list=name.split(names)

  #Variables from PCOA
  vars=pcoa.Sputum.otu
  colnames(vars)=name.list

  #Create Data set with Principal coordinates
  d=cbind(pcoa.Sputum.otu,pcoa)
  colnames(d)[8:9]=c("p1","p2")

  #Define the three clusters
  d_C1=rownames(d[which(d$p2>-.25 & d$p1 < -.1),])
  d_C2=rownames(d[which(d$p2< -0.3),])
  d_C3=rownames(d[which(d$p1>-.1 & d$p2>-.3),])

  #Obtain Alpha diversity for the clusters
  div=cbind.data.frame(diversity(rel.otu,index ="shannon"),digest)
  d_C1_div=div[d_C1,]
  d_C2_div=div[d_C2,]
  d_C3_div=div[d_C3,]
  all_C_div=cbind.data.frame(rbind(d_C1_div,d_C2_div,d_C3_div),c(rep(1,44),rep(2,4),rep(3,30)))
  colnames(all_C_div)=c("Index","Digest","Cluster")

  #plot and test
  digest_alpha_clusters=all_C_div[all_C_div$Digest=="With EnzD",]
  nondigest_alpha_clusters=all_C_div[all_C_div$Digest=="Without EnzD",]

  kruskal.test(digest_alpha_clusters$Index~digest_alpha_clusters$Cluster)

  par(mfrow=c(1,2))
  boxplot(digest_alpha_clusters$Index~digest_alpha_clusters$Cluster)
  boxplot(nondigest_alpha_clusters$Index~nondigest_alpha_clusters$Cluster)

 # extract vectors whose pvalues are less than .005
  vec.sp<-envfit(pcoa, vars)
  vec.sp.df<-as.data.frame(cbind(vec.sp$vectors$arrows*sqrt(vec.sp$vectors$r),vec.sp$vectors$pvals))
  vec.sp.df$species<-rownames(vec.sp.df)
  colnames(vec.sp.df)<-c("Dim1","Dim2","Pvals","species")
  vec.sp.df<-vec.sp.df[vec.sp.df$Pvals<.005,]


  #Pcoa plot for Sputum samples
  h=rep(1:39,each=2)
  g1<-ggplot(d,aes(x=d$p1,y=d$p2,group=h,color=digest[85:162]))+
            geom_line(col="black", linetype ="dotted")+
            geom_point(size=3)+
            guides( col=F, size=F)+
            labs(x="Component 1", y="Component 2")+
            theme(text = element_text(size=20))+
            geom_segment(data=vec.sp.df,aes(x=0,xend=Dim1,y=0,yend=Dim2),
                         arrow = arrow(length = unit(0.5, "cm")),colour="grey",
                         stat="identity",inherit.aes = FALSE) +
            geom_text(data=vec.sp.df,aes(x=Dim1,y=Dim2,label=species),
                      inherit.aes = FALSE,size=5)+
            coord_fixed()+
            xlim(-1,1)+
            ylim(-1,1)

  #Place Sputum and OP PCoA in the same figure. This is figure 1
  grid.arrange(g2,g1, ncol=2, nrow=1,heights=c( 2))

######################################## Genera  Level Comparisons ########################################

  #Wilcox Rank Sum Test --OP Only
  wilx.rel.OP.otu=wilx.rel.otu[1:84,]

  #Subset based on which taxa have a median relative abundance of at least 1%
  median.wilx=wilx.rel.OP.otu[,names(medt)]

  #Calculate absolute RA for OP swabs
  absolute.RA.OP.digest=list()
  absolute.RA.OP.non=list()
  for(i in 1:14){
        absolute.RA.OP.digest[i]=unlist(tapply(median.wilx[,i],digest[1:84],median)[2])*100
        absolute.RA.OP.non[i]=unlist(tapply(median.wilx[,i],digest[1:84],median)[1])*100
        }

  #Calculate Wilcoxon Exact Signed Rank Tests for Each taxa
  wilx.op=apply(t(median.wilx),1,function(x) wilcox.exact(x[digest_rows],x[nondigest_rows],
                                                      paired=T,conf.int = T))

  #round p-values to 2 digits
  wilx.estimate=round(unlist(lapply(wilx.op, function(x) {return(x$estimate)})),4)*100
  table.p=round(p.adjust(unlist(lapply(wilx.op, function(x){if(x$p.value){return(x$p.value)}})),method="BH"),2)
  table.p=ifelse(table.p<=0.00,0.01,table.p)
  table.p.op=ifelse(table.p==0.01,paste("<",table.p,sep=""),table.p)

  #Table One
  table.ra.op=cbind.data.frame(name.split(names(wilx.op)),
                              unlist(absolute.RA.OP.digest),
```

```
                                unlist(absolute.RA.OP.non),
                                wilx.estimate,table.p.op)

  table.ra.op=table.ra.op[-13,]
  #Name columns of Table 1
  colnames(table.ra.op)=c("Genera",
                          "EnzD %",
                          "Non-EnzD %",
                          "Estimated Change in RA %",
                          "P-Value")

  row.names(table.ra.op)<-seq(nrow(table.ra.op))

#Print Table 1
xtable(table.ra.op)




#Wilcox Rank Sum Test and Boxplots--Sputum Only
wilx.rel.Sputum.otu=wilx.rel.otu[85:162,]
median.wilx=wilx.rel.Sputum.otu[,names(meds)]

#Calculate absolute RA for sputum swabs
absolute.RA.sputum.digest=list()
absolute.RA.sputum.non=list()
for(i in 1:7){
  absolute.RA.sputum.digest[i]=tapply(median.wilx[,i],digest[85:162],median)[2]*100
  absolute.RA.sputum.non[i]=tapply(median.wilx[,i],digest[85:162],median)[1]*100
          }


#Calculate Wilcoxon Exact Signed Rank Tests for Each taxa
wilx=apply(t(median.wilx),1,
         function(x) wilcox.exact(x[digest_rows],
                                  x[nondigest_rows],
                                  paired=T,conf.int = T))

#round p-values to 2 digits
wilx.estimate=round(unlist(lapply(wilx, function(x) {return(x$estimate)})),4)*100
table.p=round(p.adjust(unlist(lapply(wilx, function(x){return(x$p.value)})),method="BH"),2)
table.p=ifelse(table.p<=0.00,0.01,table.p)
table.p.sputum=ifelse(table.p==0.01,paste("<",table.p,sep=""),table.p)

#Table One
table.ra.sputum=cbind.data.frame(name.split(names(wilx)),
                                unlist(absolute.RA.sputum.digest),
                                unlist(absolute.RA.sputum.non),
                                wilx.estimate,table.p.sputum)
#name columns in Table 1
colnames(table.ra.sputum)=c("Genera",
                          "EnzD %",
                          "Non-EnzD %",
                          "Estimated Change in RA %",
                          "P-Value")

row.names(table.ra.sputum)<-seq(nrow(table.ra.sputum))


#combine all significant bacterium for OP plus Staphylococcus (found in sputum)
#Need to be able to graph all significant taxa (OP and sputum) in one figure (Figure 3 in the paper)
significant.bacteria.genera=c(names(wilx.op)[-13],names(table.p.sputum)[2])
significant.bacteria.genera=as.character(significant.bacteria.genera)

#extract staph
staph=as.numeric(as.character(rel.OP.otu$`Bacteria/Firmicutes/Bacilli/Bacillales/Staphylococcaceae/Staphylococcus`))

#calculate p-value for staph in OP samples
staph.wilx=wilcox.exact(staph~digest[1:84],paired=T)

#combine with significant pvalues in OP samples
pvalues.bps=c(table.ra.op$`P-Value`,"0.01")
names(pvalues.bps)<-c(names(pvalues.bps),"Bacteria/Firmicutes/Bacilli/Bacillales/Staphylococcaceae/Staphylococcus")

#extract sputum pvalues for those bacterium that were significant in OP samples
sputum.vals=rel.Sputum.otu[,significant.bacteria.genera]

#Calculate p-values for staph in sputum
i=0
hold=NULL
pvalues.sputum.bps=NULL
for(i in 1:length(significant.bacteria.genera)){
                hold=wilcox.exact(sputum.vals[,i]~digest[85:162],paired=T)
                pvalues.sputum.bps[i]=hold$p.value
}

#round pvalues and replaces 0s with 0.01
round.ps=round(p.adjust(pvalues.sputum.bps,method="BH"),2)
pvals=ifelse(round.ps==0,.01,round.ps)

#reformat and attach p for graphical use
pval=ifelse(pvals=="0.01",paste("p<",pvals, sep=""),paste("p=",pvals, sep=""))
v=ifelse(nchar(pval)==5,paste(pval,"0",sep=""),pval)

#Create a new dataset with the relative abundance of all signifcant taxa based on Wilcoxon Signed Rank Tests
bps=rel.otu[,significant.bacteria.genera]
name.list1=name.split(significant.bacteria.genera)

#Create new dataframes for digest versus nondigested samples
bps.digest=bps[digest_rows,]
bps.non=bps[nondigest_rows,]

#calculate differences in relative abundance
diff.bps=bps.digest-bps.non
diff.area=factor(c(rep("OP",42),rep("Sputum",39)),ordered=F)
diff.bps=cbind(diff.bps,diff.area)
```

```
colnames(diff.bps)=c(name.list1,"area")

#Create a long format dataset to in order to use facet_wrap in ggplot; area and digest remain as Id variables
plot.dat.genera=melt(diff.bps,idvars=area)

#Figure 3
B<-ggplot(data=plot.dat.genera)+
      geom_boxplot(aes(y = plot.dat.genera$value,x=plot.dat.genera$area))+
      coord_cartesian(ylim=c(-.30,.72))+
      xlab("")+
      ylab("Genera")+
      labs(fill="Group")+
      annotate("text", x = 1, y = .73, label = pvalues.bps,size = 5) +
      annotate("text", x = 2, y = .73, label = v, size = 5)+
      facet_wrap(~variable,ncol=5)+theme(text = element_text(size=25))



##########################################  Morisita Horn Correlations ########################################

          #RA versus MH for paired samples

          dist=vegdist(otu[,3:255],method="morisita")
          #Converts from Dissimilarity matrix to a similarity matrix
          dist.mat=1-as.matrix(dist)

          #Bloack diagonal matrix for OP samples
          a<-rblock(81)
          a=as.matrix(a)

          #overlay diagonal matrix onto distance matrix
          k=as.data.frame(dist.mat*a)
          f=k[k!=0]

          #Delete duplicate MH values; only need one per pair
          mh=unique(f)

          #compute the number of MH values above .8
          m=as.data.frame(mh)
          m$group=diff.area
          sum=aggregate(m[,1]>.8, by=list(m$group),FUN=sum)
          OP.percent=sum[[2]][1]/42
          sputum.percent=sum[[2]][2]/39
          OP.percent
          sputum.percent


          #Create a boxplot for Mh values grouped by sample location
          p.value=wilcox.exact(m[,1]~m$group)$p.value

          #p.value <0.001 so format
          p.value= "p < 0.001"

          #Figure 2
          ggplot(data=m)+geom_boxplot(aes(y=mh, x = m$group),width=.42,fill = "white")+
                        ylab("MH between Pairs")+xlab("")+
                        geom_jitter(aes(y=mh, x = m$group),size=3,width = .055)+
                        scale_x_discrete(breaks=unique(m$group),
                                         labels=c("OP (n=42)","Sputum (n=39)"))+
                        theme_bw()+
                        annotate("text", x = 1.17, y = 1, label =p.value ,size = 5) +
                        theme(text = element_text(size=30),axis.line = element_line(colour = "black"),
                                      panel.grid.major = element_blank(),
                                      panel.grid.minor = element_blank(),
                                      panel.border = element_rect(colour = "black"),
                                      panel.background = element_blank())



          #calculate median realtive abundance

          meds=round(apply(rel.otu[,1:253],2,median),2)
          meds=medt[meds >=.01]
          median.wilx=rel.otu[,names(medt)]


          #use those bacteria with at least 1% relative abundance to calculate
          #correlation between digested sample RA and  MH
          nondigest.otu=median.wilx[nondigest_rows,]
          digest.otu=median.wilx[digest_rows,]

          #Create a long dataset for grouping and graphing MH against relative abundance
          bps1=cbind(nondigest.otu,factor(mh),area[nondigest_rows])
          colnames(bps1)=c(colnames(nondigest.otu),"MH","area")
          nondigest.cor.plot.dat=melt(bps1)
          nondigest.cor.plot.dat$MH=as.numeric(as.character(nondigest.cor.plot.dat$MH))

          #Figure S3B
          C<-ggplot(data=nondigest.cor.plot.dat)+
                    geom_point(aes(y=value+.001,x=MH,col=area))+
                    xlab("MH per pair")+
                    ylab("log RA for taxa")+
                    scale_y_log10()+
                    guides(col=guide_legend(title="Group"))+
                    facet_wrap(~variable,ncol=5)+
                    theme(text = element_text(size=20))

          #Calculate correlations between Mh and RA for specific taxa

           correlations=apply(digest.otu[,1:(dim(bps1)[2]-3)],
                              2,
                              FUN=cor.test,
                              y=as.numeric(as.character(bps1$MH)),
                              method="spearman")
```

```
            #grab only non digested samples
            j=seq(1,162,2)
            non.digest.otu=median.wilx[j,]

            #Gram positive bacteria nondigest
            pos.rows=c(grep("Bacteria/Firmicutes",colnames(non.digest.otu)),
                       grep("Bacteria/Actinobacteria",colnames(non.digest.otu)))
            pos.rows=pos.rows[-2]
            gram.hold=non.digest.otu[,pos.rows]
            neg.rows=c(2,3,4,8,9,11)
            gram.neg.hold=non.digest.otu[,neg.rows]
            gram.positive.nonRA=apply(gram.hold,1,sum)
            gram.negative.nonRA=apply(gram.neg.hold,1,sum)
            gram.non=as.data.frame(cbind(gram.positive.nonRA,gram.negative.nonRA))


             #Gram positive bacteria digest
            pos.rows=c(grep("Bacteria/Firmicutes",colnames(digest.otu)),
                       grep("Bacteria/Actinobacteria",colnames(digest.otu)))
            pos.rows=pos.rows[-2]
            gram.hold=digest.otu[,pos.rows]
            neg.rows=c(2,3,4,8,9,11)
            gram.neg.hold=digest.otu[,neg.rows]
            gram.positive.RA=apply(gram.hold,1,sum)
            gram.negative.RA=apply(gram.neg.hold,1,sum)
            gram.dig=as.data.frame(cbind(gram.positive.RA,gram.negative.RA))

            #Create a long dataset for grouping and graphing MH against relative abundance
            bps1=cbind(gram.non[1],factor(mh),area[j])
            colnames(bps1)=c("Gram Positive","MH","area")
            gram.non.plot.dat=melt(bps1)
            gram.non.plot.dat$MH=as.numeric(as.character(gram.non.plot.dat$MH))




            #Scatter plots Mh versus RA; Figure 3A
            g1<-ggplot(data=gram.non.plot.dat)+
                        geom_point(aes(y=gram.non.plot.dat$value,x=gram.non.plot.dat$MH,
                                       col=gram.non.plot.dat$area,
                                       size=.3))+
                        xlab("MH per pair")+
                        ylab("RA")+
                        guides(col=F,size=F)+
                        theme(text = element_text(size=30))




            #calculate differences in relative abundance
            diff.gram.pos=gram.positive.RA-gram.positive.nonRA
            diff.gram.neg=gram.negative.RA-gram.negative.nonRA

            #Create a long dataset for grouping and graphing MH against relative abundance gram positive
            diff.gram=cbind.data.frame(diff.gram.pos,mh,area[j])
            colnames(diff.gram)=c("Gram Positive","MH","area")
            pos.plot.dat.diff=melt(diff.gram, id.vars=c("MH","area"))
            pos.plot.dat.diff$MH=as.numeric(as.character(pos.plot.dat.diff$MH))

            #Figure 3B
            g2<-ggplot(data=pos.plot.dat.diff)+
                      geom_point(aes(y=pos.plot.dat.diff$value,x=pos.plot.dat.diff$MH,
                                     col=pos.plot.dat.diff$area,
                                     size=.3))+
                      xlab("MH per pair")+
                      ylab("Difference (Enzd-NonEnzd) RA")+
                      guides(col=F,size=F)+
                      theme(text = element_text(size=30))


           #Figure 3
           grid.arrange(g1,g2, ncol=2, nrow=1,heights=c( 2))

############################################## Phyla ################################################

#Create a dataset that is grouped by phyla
#Relative abundace Dataset
mytok=NULL
for(i in 1:253){mytok[i] <- paste(strsplit(colnames(rel.otu)[i],"/")[[1]][1:2],collapse="/")}

phyla.otu=rel.otu[1:253]
colnames(phyla.otu)=mytok

phyla=as.data.frame(do.call(cbind,by(t(phyla.otu),INDICES=colnames(phyla.otu),FUN=colSums)))

#Create a dataset that is grouped by phyla
#OTU Dataset
mytok=NULL
for(i in 3:255){mytok[i] <- paste(strsplit(colnames(otu)[i],"/")[[1]][1:2],collapse="/")}

mytok=na.omit(mytok)
phyla.otu=otu[,3:255]
colnames(phyla.otu)=mytok

otu.phyla=as.data.frame(do.call(cbind,by(t(phyla.otu),INDICES=colnames(phyla.otu),FUN=colSums)))


#Create a dataset that is grouped by phyla
#Dataset for use in wilcoxon exact test; excludes unclassified and Bacteria
mytok=NULL
for(i in 1:251){mytok[i] <- paste(strsplit(colnames(wilx.rel.otu)[i],"/")[[1]][1:2],collapse="/")}

mytok=na.omit(mytok)
wilx.phyla.otu=wilx.rel.otu[,1:251]
```

```
colnames(wilx.phyla.otu)=mytok

wilx.phyla=as.data.frame(do.call(cbind,by(t(wilx.phyla.otu),INDICES=colnames(wilx.phyla.otu),FUN=colSums)))


################################### Phyla Specific Comparisons ################################

#Wilcox Rank Sum Test and Boxplots--OP Only; phyla
wilx.rel.otu.OP.phyla=wilx.phyla[1:84,]

#take only those taxa whose median relative abundance are at least 1%

med=apply(wilx.rel.otu.OP.phyla[,1:17],2,median)
meds=med[med >=.01]
median.wilx.OP=wilx.rel.otu.OP.phyla[,names(meds)]

#Calculate absolute RA for OP swabs phyla
          phyla.absolute.RA.OP.digest=list()
          phyla.absolute.RA.OP.non=list()
          for(i in 1:5){
                  phyla.absolute.RA.OP.digest[i]=tapply(median.wilx.OP[,i],digest[1:84],median)[2]*100
                  phyla.absolute.RA.OP.non[i]=tapply(median.wilx.OP[,i],digest[1:84],median)[1]*100
                  }

 #Calculate Wilcoxon Exact Signed Rank Tests for Each phyla
          wilx=apply(t(median.wilx.OP),1,function(x) wilcox.exact(x[digest_rows],x[nondigest_rows],
                                                                  paired=T,conf.int = T))

          #round p-values to 2 digits
          wilx.estimate=round(unlist(lapply(wilx, function(x) {return(x$estimate)})),4)*100
          table.p=round(p.adjust(unlist(lapply(wilx, function(x){return(x$p.value)})),method="BH"),2)
          table.p=ifelse(table.p<=0.00,0.01,table.p)
          table.p=ifelse(table.p==0.01,paste("<",table.p,sep=""),table.p)

          #Table One
          table.ra.OP.phyla=cbind.data.frame(name.split(names(wilx)),
                            unlist(phyla.absolute.RA.OP.digest),
                            unlist(phyla.absolute.RA.OP.non),
                            wilx.estimate,table.p)
          #Name Table 1
          colnames(table.ra.OP.phyla)=c("Phyla",
                                        "EnzD %",
                                        "Non-EnzD %",
                                        "Estimated Change in RA %",
                                        "P-Value")
          row.names(table.ra.OP.phyla)<-seq(nrow(table.ra.OP.phyla))


#Print Table 1
xtable(table.ra.OP.phyla)

#wilcox Exact test for phyla in OP samples
wilx=apply(t(median.wilx.OP),1,function(x) wilcox.exact(x[digest_rows],x[nondigest_rows],paired=T,conf.int=T))
raw.p=round(unlist(lapply(wilx, function(x) if(x$p.value < .05){return(x$p.value)})),2)
estiamtes=round(unlist(lapply(wilx, function(x) {return(x$estimate)})),4)
phyla.wilx.OP=ifelse(raw.p<=0.00,0.01,raw.p)

#Wilcox Rank Sum Test and Boxplots--Sputum Only; phyla
wilx.rel.otu.Sputum.phyla=wilx.phyla[85:162,]

#take only those taxa whose median relative abundance are at least 1%
med=apply(wilx.rel.otu.Sputum.phyla[,1:16],2,median)
meds=med[med >=.01]
median.wilx.sputum=wilx.rel.otu.Sputum.phyla[,names(meds)]

#Calculate absolute RA for sputum swabs phyla
          phyla.absolute.RA.sputum.digest=list()
          phyla.absolute.RA.sputum.non=list()

          for(i in 1:4){
                  phyla.absolute.RA.sputum.digest[i]=tapply(median.wilx.sputum[,i],digest[85:162],median)[2]
                  phyla.absolute.RA.sputum.non[i]=tapply(median.wilx.sputum[,i],digest[85:162],median)[1]
          }


          phyla.table.ra.sputum=cbind(phyla.absolute.RA.sputum.non,
                                      phyla.absolute.RA.sputum.digest)

          colnames(phyla.table.ra.sputum)=c("Non-EnzD",
                                            "EnzD")

 #Calculate Wilcoxon Exact Signed Rank Tests for Each phyla
          wilx=apply(t(median.wilx.sputum),1,
                     function(x) wilcox.exact(x[digest_rows],
                                              x[nondigest_rows],
                                              paired=T,conf.int = T))

          #round p-values to 2 digits
          wilx.estimate=round(unlist(lapply(wilx, function(x) {return(x$estimate)})),4)*100
          table.p=round(p.adjust(unlist(lapply(wilx, function(x){return(x$p.value)})),method="BH"),2)
          table.p=ifelse(table.p<=0.00,0.01,table.p)
          table.p=ifelse(table.p==0.01,paste("<",table.p,sep=""),table.p)

          #Table One
          table.ra.sputum.phyla=cbind.data.frame(name.split(names(wilx)),
                            unlist(phyla.absolute.RA.sputum.digest),
                            unlist(phyla.absolute.RA.sputum.non),
                            wilx.estimate,table.p)
          #Name Table 1
          colnames(table.ra.sputum.phyla)=c("Phyla",
                                            "EnzD %",
                                            "Non-EnzD %",
                                            "Estimated Change in RA %",
                                            "P-Value")
          row.names(table.ra.sputum.phyla)<-seq(nrow(table.ra.sputum.phyla))
```

```
#Print Table 1
xtable(table.ra.sputum.phyla)

##wilcox Exact test for phyla in sputum samples
wilx=apply(t(median.wilx.sputum),1,function(x) wilcox.exact(x[digest_rows],x[nondigest_rows],paired=T,conf.int=T))
phyla.sputum.estimates=raw.p=round(unlist(lapply(wilx, function(x) {return(x$estimate)})),2)
raw.p=round(unlist(lapply(wilx, function(x) if(x$p.value < .05){return(x$p.value)})),2)
phyla.wilx.sputum=ifelse(raw.p<=0.00,0.01,raw.p)

significant.bacteria.phyla=as.character(names(phyla.wilx.OP))
significant.bacteria.phyla=as.character(significant.bacteria.phyla)
pvalues.bps=phyla.wilx.OP

#calculate Staph p-values as before
sputum.vals=wilx.rel.otu.Sputum.phyla[,significant.bacteria.phyla]

i=0
hold=NULL
pvalues.sputum.bps=NULL

for(i in 1:length(significant.bacteria.phyla)){
  hold=wilcox.exact(sputum.vals[,i]~digest[85:162],paired=T)
  pvalues.sputum.bps[i]=hold$p.value
}

round.ps=round(pvalues.sputum.bps,2)
pvals=ifelse(round.ps==0,.01,round.ps)
pval1=ifelse(pvals==.01,paste("p<",pvals, sep=""),paste("p=",pvals, sep=""))


#Create a new dataset with the relative abundance of all signifcant taxa based on Wilcoxon Signed Rank Tests

bps=wilx.phyla.otu[,significant.bacteria.phyla]


#format names and p-values
name.list1=name.split(significant.bacteria.phyla)
name.pval1=ifelse(pvalues.bps==.01,paste("p<",pvalues.bps, sep=""),paste("p=",pvalues.bps, sep=""))

#Create data frames for digested versus non digested
bps=cbind(bps,area,digest)
colnames(bps)=c(name.list1,"area","digest")
bps.digest=bps[bps$digest=="With EnzD",]
bps.non=bps[bps$digest=="Without EnzD",]

#Calculate difference in RA between digested and non digested
diff.bps=bps.digest[,1:5]-bps.non[,1:5]
diff.area=factor(c(rep("OP",42),rep("Sputum",39)))
diff.bps=cbind(diff.bps,diff.area)

#Create a long format dataset to in order to use facet_wrap in ggplot; area and digest remain as Id variables
plot.dat1=melt(diff.bps,idvars=diff.area)

#Figure 3A
A<-ggplot(data=plot.dat1)+
        geom_boxplot(aes(y = value,x=factor(diff.area)))+
        coord_cartesian(ylim=c(-.30,.55))+
        xlab("")+
        ylab("Phyla")+
        labs(fill="Group")+
        annotate("text", x = 1, y = .55, label = name.pval1,size = 5) +
        annotate("text", x = 2, y = .55, label = pval1,size = 5)+
        facet_wrap(~variable, nrow = 4, ncol = 5)+
        theme(text = element_text(size=25))

#figure 3
grid.arrange(A, B, ncol=1, nrow=2,heights=c(.8, 2))

#stacked bar chart for significant phyla in OP samples
names=names(phyla.wilx.OP)


med=apply(wilx.phyla[,1:17],2,median)
meds=med[med >=.01]
median.wilx=wilx.phyla[,names(meds)]


#################################### MH Correlations in Phyla ########################################


#grab only non digested samples
j=seq(1,162,2)
non.digest.otu=median.wilx[j,]

#Create a long dataset for grouping and graphing MH against relative abundance
bps1=cbind(non.digest.otu,factor(mh),area[j])
colnames(bps1)=c(colnames(non.digest.otu),"MH","area")
phyla.cor.plot.dat=melt(bps1)
phyla.cor.plot.dat$MH=as.numeric(as.character(phyla.cor.plot.dat$MH))


#Calculate correlations between Mh and RA for specific taxa
bps1$MH=as.numeric(as.character(bps1$MH))


c=list()
name=NULL
i=0


for(i in 1:(dim(bps1)[2]-3)){
  if(cor.test(non.digest.otu[,i],bps1$MH,method="spearman")$p.value<= 0.05){
    c[[i]]=cor.test(non.digest.otu[,i],bps1$MH,method="spearman")
    name[i]=colnames(non.digest.otu)[i]
  }}
```

```
#Figure S3A
D<-ggplot(data=phyla.cor.plot.dat)+
        geom_point(aes(y=value+.001,x=MH,col=area))+
        xlab("MH per pair")+
        ylab("log RA for taxa")+
        scale_y_log10()+
        guides(col=guide_legend(title="Group"))+
        facet_wrap(~variable,ncol=5)+
        theme(text = element_text(size=20))


#Figure S3
grid.arrange(D, C, ncol=1, nrow=2,heights=c(.8, 2))

###################################### Alpha Diversity Plot: Supplement ##############################

#Create Dataframes for digested versus non digested
rel.otu=cbind.data.frame(rel.otu,digest)
digest.otu=rel.otu[rel.otu$digest=="With EnzD",]
non.digest.otu=rel.otu[rel.otu$digest=="Without EnzD",]

#Create distance matrices from digested and non digested dataframes
digest.Odiv=diversity(digest.otu[,1:253],index="shannon")
non.Odiv=diversity(non.digest.otu[,1:253],index="shannon")
Ddiv=digest.Odiv-non.Odiv

#area variables
diff.area=factor(c(rep(1,42),rep(2,39)),levels=c(1,2),labels=c("OP","Sputum"))

#long format data frame
diversity.plot.dat=cbind.data.frame(Ddiv,mh,diff.area)

#plot Figure S2
ggplot(diversity.plot.dat)+
        geom_point(aes(x=mh,y=Ddiv,
                       col=factor(diff.area),
                       size=.3))+
        guides(col=guide_legend(title="Sample Type",
                                override.aes=list(size=5)),
                                size=F)+
        xlab("Morisita Horn")+
        ylab("Difference in Alpha Diversity (EnzD-nonEnzD)")+
        theme(text = element_text(size=33))


###################################### Table 3 ##################################################

library("xtable")

#Load in excel file of CF pathogens from Explicet
pathogens=read.csv("C:/Users/Connie/Desktop/CF_pathogens.csv",header=T)

#Format the raw file
pathogens=cbind.data.frame(apply(pathogens,2,function(x){gsub("%","",x)}))
pathogens=otu_from_explicet(pathogens)

#Create pseudomonas plus
pathogens$pseudomonasplus=pathogens$`Bacteria/Proteobacteria/Gammaproteobacteria/Pseudomonadales`+
  pathogens$`Bacteria/Proteobacteria/Gammaproteobacteria/Pseudomonadales/Pseudomonadaceae/Pseudomonas`

#Subset into digested and non-digested samples
digest=pathogens[grepl("B",pathogens$Id),]
non_digest=pathogens[!(pathogens$Id%in%digest$Id),]

#Calculate the difference in RA between digested and non-digested samples
difference_data=cbind.data.frame(non_digest$Id,digest[,2:8]-non_digest[,2:8])
colnames(difference_data)=c("Id",colnames(digest)[2:8])

#separate the differences by sample type
op=difference_data[grepl("C",difference_data$Id),]
sputum=difference_data[grepl("E",difference_data$Id),]



#Extract any differences greater than 1%
op_Haem_increase=op[op$`Bacteria/Proteobacteria/Gammaproteobacteria/Pasteurellales/Pasteurellaceae/Haemophilus`>1,4]
op_Haem_decrease=op[op$`Bacteria/Proteobacteria/Gammaproteobacteria/Pasteurellales/Pasteurellaceae/Haemophilus`< -1,4]

op_staph_increase=op[op$`Bacteria/Firmicutes/Bacilli/Bacillales/Staphylococcaceae/Staphylococcus`>1,2]
op_staph_decrease=op[op$`Bacteria/Firmicutes/Bacilli/Bacillales/Staphylococcaceae/Staphylococcus`< -1,2]

op_Achrom_increase=op[op$`Bacteria/Proteobacteria/Betaproteobacteria/Burkholderiales/Alcaligenaceae/Achromobacter` >1,3]
op_Achrom_decrease=op[op$`Bacteria/Proteobacteria/Betaproteobacteria/Burkholderiales/Alcaligenaceae/Achromobacter`< -1,3]

op_steno_increase=op[op$`Bacteria/Proteobacteria/Gammaproteobacteria/Xanthomonadales/Xanthomonadaceae/Stenotrophomonas`>1,7]
op_steno_decrease=op[op$`Bacteria/Proteobacteria/Gammaproteobacteria/Xanthomonadales/Xanthomonadaceae/Stenotrophomonas` < -1,7]

op_pseud_increase=op[op$pseudomonasplus>1,8]
op_pseud_decrease=op[op$pseudomonasplus < -1,8]

sputum_Haem_increase=sputum[sputum$`Bacteria/Proteobacteria/Gammaproteobacteria/Pasteurellales/Pasteurellaceae/Haemophilus`>1,4]
sputum_Haem_decrease=sputum[sputum$`Bacteria/Proteobacteria/Gammaproteobacteria/Pasteurellales/Pasteurellaceae/Haemophilus`< -1,4]

sputum_staph_increase=sputum[sputum$`Bacteria/Firmicutes/Bacilli/Bacillales/Staphylococcaceae/Staphylococcus`>1,2]
sputum_staph_decrease=sputum[sputum$`Bacteria/Firmicutes/Bacilli/Bacillales/Staphylococcaceae/Staphylococcus`< -1,2]

sputum_Achrom_increase=sputum[sputum$`Bacteria/Proteobacteria/Betaproteobacteria/Burkholderiales/Alcaligenaceae/Achromobacter` >1,3]
sputum_Achrom_decrease=sputum[sputum$`Bacteria/Proteobacteria/Betaproteobacteria/Burkholderiales/Alcaligenaceae/Achromobacter`< -1,3]

sputum_steno_increase=sputum[sputum$`Bacteria/Proteobacteria/Gammaproteobacteria/Xanthomonadales/Xanthomonadaceae/Stenotrophomonas`>1,7]
sputum_steno_decrease=sputum[sputum$`Bacteria/Proteobacteria/Gammaproteobacteria/Xanthomonadales/Xanthomonadaceae/Stenotrophomonas` < -1,7]

sputum_pseud_increase=sputum[sputum$pseudomonasplus>1,8]
sputum_pseud_decrease=sputum[sputum$pseudomonasplus< -1,8]
```

```
#Create a list

bacteria_increase=list(op_Haem_increase,op_staph_increase,op_Achrom_increase,op_steno_increase,op_pseud_increase,
                       sputum_Haem_increase,sputum_staph_increase,sputum_Achrom_increase,sputum_steno_increase,sputum_pseud_increase)

bacteria_decrease=list(op_Haem_decrease,op_staph_decrease,op_Achrom_decrease,op_steno_decrease,op_pseud_decrease,
                       sputum_Haem_decrease,sputum_staph_decrease,sputum_Achrom_decrease,sputum_steno_decrease,sputum_pseud_decrease)

#Find summary statistics
IQR_increase=do.call(rbind, lapply(bacteria_increase,summary))

IQR_decrease=do.call(rbind, lapply(bacteria_decrease,summary))
```
